# Presumed Bartonella-Associated Spondylodiscitis in a 3-Year-Old Child: A Case Report and Review of the Literature

**DOI:** 10.3390/children12050649

**Published:** 2025-05-16

**Authors:** Hadi El Assaad, Eckehard Schumann, Christian Klemann, Nadine Dietze-Jergus, Christoph-Eckhard Heyde, Philipp Pieroh

**Affiliations:** 1 Department of Orthopedics, Trauma and Plastic Surgery, University of Leipzig, 04103 Leipzig, Germany; eckehard.schumann@medizin.uni-leipzig.de (E.S.); christoph-eckhard.heyde@medizin.uni-leipzig.de (C.-E.H.); philipp.pieroh@medizin.uni-leipzig.de (P.P.); 2 Department of Pediatric Immunology, Rheumatology and Infectiology, Hospital for Children and Adolescents, University of Leipzig, 04103 Leipzig, Germany; christian.klemann@medizin.uni-leipzig.de; 3 Institute of Medical Microbiology and Virology, University of Leipzig, 04103 Leipzig, Germany; nadine.dietze@medizin.uni-leipzig.de

**Keywords:** spondylodiscitis, vertebral osteomyelitis, *Bartonella henselae*, cat scratch disease, bone involvement, case report

## Abstract

With an incidence of 0.3 per 100,000, spondylodiscitis is a rare condition in children. It is typically bacterial in origin and most commonly caused by *Staphylococcus aureus*. Bone involvement in cat-scratch disease (CSD) due to *Bartonella henselae* is exceedingly rare, occurring in only 0.17–0.27% of cases. We present the case of a 3-year-old boy with a two-week history of intermittent back pain and a recent onset fever. Initial laboratory findings were unremarkable, and MRI revealed spondylodiscitis at L3/4 without abscess formation. Empirical antibiotic treatment with ampicillin/sulbactam showed no clinical response. Serologic testing revealed a positive *Bartonella henselae* IgM (IgG negative), leading to a change in antibiotic treatment to azithromycin and rifampicin for three weeks, resulting in rapid clinical improvement. Follow-up at nine weeks showed marked clinical and radiologic improvement. Although IgM subsequently turned negative without IgG seroconversion—a pattern previously described in *Bartonella* infections—this does not exclude the diagnosis. Biopsy or tissue PCR was not performed due to the mild clinical course. A review of the literature identified 28 pediatric cases of *Bartonella henselae* spondylodiscitis, with significant variation in diagnostic and treatment approaches. This case underscores the importance of considering *Bartonella* in the differential diagnosis of pediatric vertebral osteomyelitis.

## 1. Introduction

Spondylodiscitis is the infection of the intervertebral disc and the adjacent vertebrae [1,2]. Compared to adults, it is rare in children with an incidence of 0.3 versus 2.4 per 100,000 patients, respectively. Moreover, it accounts for only 3% of osteoarticular infections in pediatric hospitals [1,3].

In children, spondylodiscitis is primarily bacterial, with *Staphylococcus aureus* as the most common pathogen. *Kingella kingae* has also been recognized as a significant pathogen in pediatric patients, along with *Streptococci* [4]. Rare causes of pediatric spondylodiscitis include fungal pathogens such as *Candida *spp. and *Aspergillus *spp., parasitic infections like *Echinococcus *spp. [4] as well as cat-scratch disease, caused by *Bartonella henselae*.

Members of the genus *Bartonella* are fastidious, pleomorphic, Gram-negative rods belonging to the α2 subgroup of Proteobacteria, closely related to genera such as *Brucella*, *Agrobacterium*, and *Rhizobium* [5]. The species responsible for most human infections are *Bartonella henselae*, *B. quintana*, and *B. bacilliformis* [6].

Cat-scratch disease (CSD) is primarily characterized by subacute, localized, self-limiting lymphadenitis, typically preceded by a local cutaneous reaction at the scratch site. In 90% of cases, symptoms resolve within 2–4 weeks without antibiotic treatment [7]. However, about 10% of cases present with atypical manifestations such as prolonged fever, erythema nodosum, and granulomas in the liver or spleen. Bone involvement in CSD is extremely rare, reported in only 0.17–0.27% of cases [7,8]. Additionally, vertebral involvement accounts for 42% of bone-related CSD cases [9].

We present a pediatric case of spondylodiscitis caused by CSD, an exceptionally rare condition, along with a review of the literature. This case contributes valuable insight to the limited body of knowledge on CSD-induced vertebral involvement [4,10].

## 2. Case Presentation

**Figure 1:**
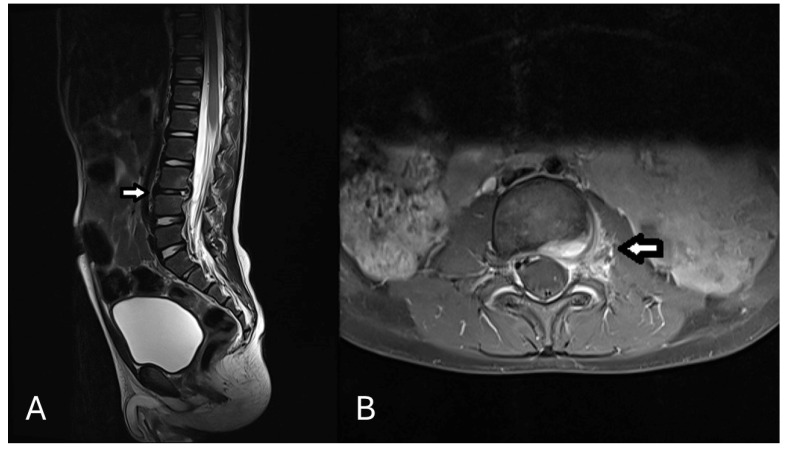
Initial MRI showing the spondylodiscitis L3/4. (**A**): Contrast enhanced MRI T2 Sagittal sequence at presentation showing a reduced height and decreased T2 signal of the intervertebral disc at L3/4. No bony erosions, vertebral body destruction, or facet joint involvement was detected. (**B**): Contrast enhanced MRI T1 transversal sequence at presentation showing the edema of the affected disc.

A 3-year-old male patient without a previous medical history was admitted to the hospital with a two-week history of intermittent back pain. Before pain onset he sustained a spinal contusion at kindergarten, and a fracture was ruled out. He reported a mild decrease in pain. Since then, the patient was occasionally observed limping while walking. In addition, the parents reported episodes of fever (up to 39 °C) that started three days before admission. His pediatrician could not determine the site of infection. The patient resides in the eastern region of Germany, and the parents denied recent travel. According to the parents, the patient had contact with cats 6–10 weeks prior to presentation but has not sustained any open wounds or scratches. On clinical examination, he had normal posture with no spinal deformities or other abnormal findings. Routine blood investigations revealed normal levels of C-reactive protein (CRP) at 1.78 mg/L (reference value  <  5 mg/L) and non-pathological leucocytes count of 8 × 10^9^/L (reference: 5–12 × 10^9^/L). A hip ultrasound examination bilaterally revealed no fluid collection or other joint abnormalities. A lumbar spine and pelvis magnetic resonance imaging (MRI) showed a focal spondylodiscitis L 3/4 without evidence of abscess formations (Figure 1). An abdominal sonography was performed without evidence for infectious focus or organomegaly.

Other infectious foci were excluded. An intravenous broad spectrum antibiotic therapy with ampicillin and sulbactam as well as analgesic therapy was initiated. The initial blood cultures were negative. Extensive infectious diagnostic workup including serology for *Borrelia* and *Bartonella* and an Interferon Gamma Release Assay (IGRA)-Test for tuberculosis were performed. On day 8 of hospitalization, *Bartonella henselae* serology was positive for IgM (IgG negative) through immunofluorescence assay, while all other infectious tests were negative, prompting a change in antibiotic treatment to the combination of azithromycin (75 mg daily) and rifampicin (150 mg daily). Oropharyngeal sample collection was initially planned to test for *Kingella kingae*. However, after receiving positive serology results for *Bartonella henselae* established the microbiological diagnosis, the *Kingella kingae* PCR was not performed. Furthermore, a corset to immobilize the spine was fitted and worn by the patient during the hospitalization. After starting antibiotic therapy and the spine immobilization, the patient showed a rapid clinical improvement, and the pain quickly subsided.

On day 12 of hospitalization, the patient was discharged on oral antibiotics (azithromycin and rifampicin). Antibiotic treatment was administered intravenously for one week, followed by two weeks orally. Spinal immobilization using the corset was recommended for a further six weeks, and a control MRI was planned after 8–12 weeks. Serology was repeated two weeks after initial diagnosis and showed IgM positivity further without evidence for IgG. In accordance with current recommendations, we performed an additional PCR on blood, which was negative.

**Figure 2:**
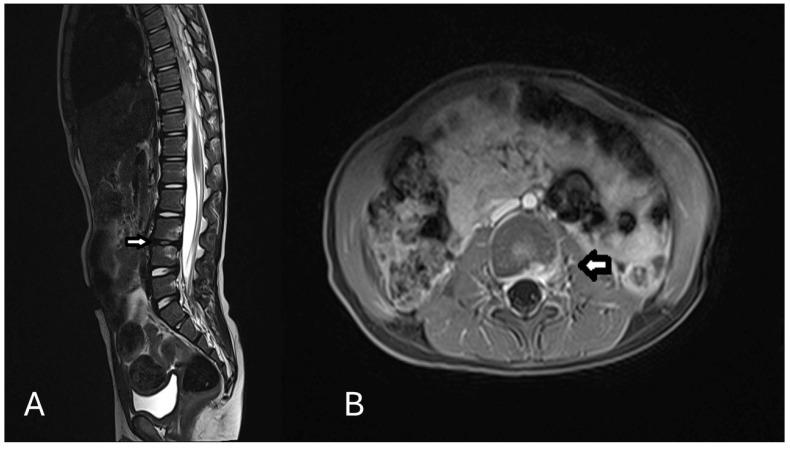
(**A**): T2 Sagittal sequence and (**B**): T1 transversal sequence of the control contrast enhanced MRI after 9 weeks showing significant regression of the inflammatory contrast agent uptake dorsolaterally in the intervertebral disc space at L3/4.

After nine weeks, the follow-up MRI showed marked regression of inflammatory changes and a substantial reduction in disease activity (Figure 2). Serology testing was repeated without evidence of IgG and IgM positivity. Although this serological course does not fulfil classical criteria for confirmed infection, it has been previously described in *Bartonella* infections and does not exclude the diagnosis. Due to the mild clinical course, a biopsy or PCR from tissue was not performed. The laboratory, radiological and clinical examination all indicated a rapid improvement.

## 3. Literature Review

We conducted a literature review in PubMed for other case reports or case series of pediatric cases with *Bartonella henselae* vertebral osteomyelitis (Table 1). We found a total of 18 publications (2 case series, 16 case reports). We excluded non-pediatric and non-vertebral osteomyelitis cases. In those papers overall 28 patients were reported.

The median age was 7 years (range: 2–15), and regarding the location of osteomyelitis, 7 cases had cervical, 15 had thoracic, 10 had lumbar, and 6 had sacral involvement. Nearly all cases used *Bartonella henselae* serology (27 cases) and MRI (25 cases) for the diagnosis. Biopsies and aspirates were used in 12 cases followed by PCR of the specimen. Biopsies did not reveal additional information. Regarding the antibiotic treatment, it varied between the cases. Rifampicin was the most commonly used (17 cases), followed by azithromycin (10 cases), doxycycline (8 cases), trimethoprim/sulfamethoxazole (TMP/SMZ; 6 cases), ciprofloxacin (5 cases), and gentamycin (4 cases). Surgery was needed in two cases where antibiotic treatment did not lead to improvement. (atlantoaxial fusion [13], surgical drainage of the abscess and a laminectomy at C3–C5 [21]).

## 4. Discussion

We present a case of a 3-year-old boy who presented with back pain and fever diagnosed with *Bartonella henselae*-induced spondylodiscitis. The diagnosis was established after a series of tests, including MRI, blood cultures and serology. The diagnosis was supported by serology and MRI. The patient was treated with an antibiotic regimen and responded well.

Bone involvement is a rare manifestation of *Bartonella henselae*, with spondylodiscitis being even more uncommon [7,9].

The patient’s initial symptoms of fever and back pain are non-specific. Though MRI remains the gold standard for the radiological visualization of spondylodiscitis (92% sensitivity and 96% specificity), it does not identify the underlying cause [28].

Blood cultures are performed first to detect the most common infectious causes of spondylodiscitis, including *Staphylococcus aureus* [28]. Tuberculosis can be ruled out using the tuberculin skin test or the Interferon Gamma Release Assay (IGRA) [29]. In our case, the IGRA was used. When the results of these initial tests were negative, a more extensive infectious disease.

There are many diagnostic tools to detect *Bartonella henselae*. Blood cultures are usually negative. PCR is highly specific and sensitive for detecting *Bartonella henselae* DNA in pus or lymph node specimens. In the case of spondylodiscitis, obtaining such samples is not always feasible [30,31].

Immunofluorescence assays (IFA), enzyme-linked immunosorbent assays (ELISA), and chemiluminescence immunoassays (CLIA) can all be used to detect *Bartonella* from serological samples [32,33,34]. In our case, chemiluminescence immunoassay was performed using VirClia^®^ (which has a specificity of 98.2% for predicting IgM results [34]), and detected the infectious agent, as IgM was positive for *Bartonella henselae* on presentation and again after 2 weeks. In the serology follow-up after 9 weeks IgM was negative probably due to the regression of the infection, while IgG remained negative possibly because of a delayed or absent seroconversion [32,35].

In our case, the exact mechanism by which *Bartonella henselae* led to spondylodiscitis was not clear. Previous case reports on *Bartonella henselae* with bone involvement suggested that it may occur as a direct extension from an infected lymph node [36] or via hematogenous spread from the initial inoculation site (mainly skin) or infected lymph node [37]. But in our patient, no affected lymph node or skin injury was detected.

The patient was treated with a prolonged course of azithromycin, the standard therapy for *Bartonella henselae* infections [38]. Rifampicin was added to the regimen based on its frequent use in reported cases of *Bartonella henselae* vertebral osteomyelitis and its demonstrated efficacy. A retrospective study of 268 patients with mild to severe cat-scratch disease found rifampicin to be among the most effective agents, alongside ciprofloxacin, gentamicin, and TMP/SMZ [39]. Considering these findings and given the potential synergistic effect of rifampicin and azithromycin in treating osteomyelitis [40], we opted for this combination.

Moreover, no surgical intervention was needed in this case, and the patient responded well to non-operative management. As outlined in our literature review, the majority of cases responded well to non-operative antibiotic treatment. We acknowledge that antibiotic therapy was initiated empirically before the completion of all microbiological investigations, which is generally not recommended according to adult guidelines [41]. However, no pediatric-specific spondylodiscitis guidelines currently exist. Although the Pediatric Infectious Diseases Society (PIDS) and the Infectious Diseases Society of America (IDSA) recommend invasive diagnostics to establish a microbiological diagnosis, they also emphasize that the decision must consider factors such as procedural and sedation risks [42]. In this case, the patient was clinically stable, had not received prior antibiotics, and non-invasive diagnostic tests were still pending. Given these considerations, we decided to initiate empirical antibiotic therapy. Although cephalosporins are recommended as first-line therapy in regions with low MRSA prevalence, we selected ampicillin/sulbactam based on our institutional antibiotic guidelines, which consider rising resistance rates, particularly among *E. coli*, and regional susceptibility patterns [43].

*Bartonella henselae* systemic involvement is a very rare presentation. The few pediatric cases that do exist describe diagnostic delays due to the unusual presentation, though successful outcomes were reported with appropriate antibiotics [44]. Bone involvement is not the only systemic involvement of CSD. In fact, it has also been described that CSD can affect the eyes [45], heart [46], nervous system [47] and hepatosplenic system [48,49]. Therefore, if there is no pathogen detected, *Bartonella henselae* should be considered.

The patient had a favourable prognosis following treatment, with resolution of symptoms. The overall prognosis for pediatric spondylodiscitis is, in general, good, particularly when treated early [9]. However, Bartonella-induced spondylodiscitis requires careful follow-up to monitor for potential complications as mentioned above, which were fortunately not observed in this patient [28].

In conclusion, this case highlights a presumed *Bartonella henselae* -associated spondylodiscitis in a young child and underscores the importance of considering Bartonella species in the differential diagnosis of atypical osteoarticular infections in children—even in the absence of lymphadenopathy or serologic confirmation—particularly when classical pathogens do not respond to standard therapy. A rapid clinical response to targeted antibiotic therapy can support the presumptive diagnosis in the absence of confirmatory biopsy.

## Figures and Tables

**Table 1:** Literature review of pediatric cases with *Bartonella henselae* vertebral osteomyelitis. CSD: Cat Scratch Disease, MRI: Magnetic resonance imaging, PET/CT: positron emission tomography/computed tomography scan, CT: Computed Tomography, PCR: Polymerase chain reaction, US: Ultrasound, SPECT: Single-photon emission computed tomography, TMP/SMZ: Trimethoprim/sulfamethoxazole, PO: Per Os, IV: Intravenous, OD: Once daily, BID: twice daily, Q8: every 8 h, d: days, w: weeks, m: months, * case series (13 cases, 10 cases with spinal involvement) imaging studies performed for all 13 patients: 8 MRI 2 bone scan 1 CT 2 plain radiograph.

	Year	Age (Years)	Location of Osteomyelitis	CSD Diagnosis	Management Plan
Topçu et al. [11]	2024	15	Lumbar vertebral	- MRI - right axillary biopsy - PET/CT - *Bartonella henselae* serology	- azithromycin - followed by teicoplanin + cefotaxime added to azithromycin - then rifampicin was added (4 w of hospitalization) - then ciprofloxacin + azithromycin + rifampicin PO (home)
Abu-Rumeileh et al. [12]	2022	10	Lumbar vertebral	- MRI - *Bartonella henselae* serology - CT-guided percutaneous biopsy	- doxycycline (150 mg) + rifampicin (600 mg) - then home antibiotics PO (4.5 w)
Mathkour et al. [13]	2022	2	Cervical vertebral	- CT - MRI - *Bartonella henselae* titers - bone culture	- surgery with atlantoaxial fusion - vancomycin and piperacillin–tazobactam (post-op) - then azithromycin + rifampicin PO (6 w)
Erdem et al. [14]	2018 *	5	Cervical, thoracic and sacral vertebral + femur + tibia	- *Bartonella henselae* serology - LN aspirate PCR - Biopsy	- rifampicin (3 w) + doxycycline (9 w)
		12	Thoracic vertebral + sacrum	- *Bartonella henselae* serology	- rifampicin + doxycycline (2 w)
		7	Lumbar vertebral (L2)	- *Bartonella henselae* serology	- azithromycin (2 w)
		5	Thoracic vertebral	- *Bartonella henselae* serology - Inflammatory region aspirate PCR - Biopsy	- rifampicin (3.5 w) + doxycycline (6 w)
		7	Thoracic and sacral vertebral	- *Bartonella henselae* serology	- TMP/SMZ (15 d) + rifampicin (5 w) + ciprofloxacin (5 w)
		3	Lumbar vertebral	- *Bartonella henselae* serology - bone PCR - Biopsy	- TMP/SMZ (2 w) + rifampicin (10 d) + ciprofloxacin (30 d)
		5	Thoracic vertebral (T3)	- *Bartonella henselae* serology	- azithromycin (4 w) + clindamycin (1 d)
		5	Lumbar vertebral (L2)	- *Bartonella henselae* serology	- azithromycin (1 w)
		10	Entire spine	- *Bartonella henselae* serology	- rifampicin (50 d) + doxycycline (50 d) + prednisone (2 w)
		10	Sacral vertebral (S3-4) + femur	- *Bartonella henselae* serology - Biopsy	- rifampicin (3 w) + Doxycycline (3 w)
Akbari et al. [15]	2018	7	Cervical vertebral (C3) paravertebral abscess (C2-4)	- MRI - CT guided fine-needle aspiration - *Bartonella henselae* serology	- azithromycin + rifampicin (6 w)
Kopsidas et al. [16]		11	Thoracic vertebral + paraspinal abscess	- MRI - *Bartonella henselae* serology	- gentamicin + doxycycline IV (2 w) - rifampicin + doxycycline PO (10 w)
Rafferty et al. [17]	2017	5	Vertebral (C7-T2)	- MRI - *Bartonella henselae* serology - Abdominal US	- ciprofloxacin (250 mg) IV BID + rifampicin (300 mg) PO (6 w)
Dornbos et al. [18]	2016	5	Thoracic vertebral (T8)	- MRI - *Bartonella henselae* Serology - Percutaneous image-guided vertebral biopsy then PCR	- azithromycin - then doxycycline + rifampicin (IV then PO) (6–8 w)
Zepeda et al. [19]	2016	8	Thoracolumbar Vertebral	- Abdominal US - MRI - *Bartonella henselae* serology	- clarithromycin 15 mg/kg PO BID (6 w) + ciprofloxacin IV (2 w)
Al-Rahawan [20]	2012	7	Thoracic vertebral (T5 -T9)	- MRI - A thoracoscopic incisional biopsy + PCR - *Bartonella henselae* serology	- azithromycin PO (2 w)
Tasher et al. [21]	2009	5	Cervical vertebral	- *Bartonella henselae* serology - CT - MRI	- gentamycin (parenteral) + rifampicin (PO) for 4 w - then, rifampicin + azithromycin (PO) (6 w)
Hussain et al. [22]	2007	3	Thoracolumbar vertebral	- Bone scan - MRI - CT - *Bartonella henselae* serology - PCR abscess fluid	- cefazolin IV - afterwards clindamycin + gentamicin IV (8 w) - then TMP/SMZ (4 w)
Vermeulen et al. [10]	2006	9	Cervical vertebral	- Bone scan - MRI - PCR on open biopsy - *Bartonella henselae* serology	- amoxicillin/clavulanic acid IV (3 w)
De Kort et al. [23]	2006	9	Lumbosacral vertebral, multifocal osteomyelitis	- Bone scan - MRI - PCR on bone biopsy	- rifampicin (600 mg) OD + TMP/SMZ (480 mg) BID (6 w)
Abdel-Haq et al. [24]	2005	5	Thoracic vertebral (T4-7)	- MRI - CT - *Bartonella henselae* serology	- ceftriaxone + vancomycin IV - surgical resection of epidural mass - clarithromycin PO then TMP/SMZ PO (10 w)
Santo et al. [25]	2002	2	Lumbar vertebral (L4-5)	- MRI - *Bartonella henselae* serology	- ceftriaxone + cephradine - then azithromycin (10 mg/kg/d)
		2	Lumbar vertebral (L2-3)	- Scintigraphy - MRI - *Bartonella henselae* serology	- teicoplanin + ceftriaxone (2 w) - cefaclor PO (15 d)
Pocheville et al. [26]	2002	12	Thoracic vertebral	- *Bartonella henselae* serology - MRI - SPECT	- erythromycin (500 mg) PO Q8 (2 m)
Robson et al. [27]	1999	9	Thoracic vertebral (T9) + paravertebral mass	- MRI - PCR from vertebral column aspirate - *Bartonella henselae* serology	- gentamicin (6 mg/kg/d) (8 d) + rifampicin (80/200 mg BID) + TMP/SMZ (300 mg BID) (12 w)

## Data Availability

No new data were created or analyzed in this study. Data sharing is not applicable to this article.
